# Short Medical Trip to Ghana: A Reality Check

**DOI:** 10.4269/ajtmh.19-0203

**Published:** 2019-11

**Authors:** Sangita Basnet

**Affiliations:** Department of Pediatrics, St. John’s Children’s Hospital, Southern Illinois University School of Medicine, Springfield, Illinois

One of the painful aspects of global health and short medical visits is seeing cases for which you have nothing to offer. How do you say to a mother of a 10-month-old baby girl with giant congenital melanocytic nevus that she does not have many options? The irregular, hyperpigmented, rough, keratotic lesion almost covered her back and was confluent with the lesion on her lower abdomen, covering her perineum and large circumferential areas of her thighs. Not knowing whether she could even afford it, we could only advise her to go to Accra, the capital and largest city in Ghana, to see a specialist.

I have some experience working in low-resource settings. I visit Nepal twice a year to work and teach in the pediatric intensive care unit (PICU) at Patan Hospital, a public hospital in Kathmandu. I helped establish Patan Hospital’s six-bed PICU and a six-bed neonatal intensive care unit in 2009. At that time, 22 international experts, myself included, visited Patan Hospital over 3 months on a rotating basis to train 25 physicians and 60 nurses. Through this work, I often face similar heartbreaking scenarios, where I know I could make a difference had the child been in the United States; however, because of lack of resources and finances in Nepal, I may not have much to offer.

This trip was my first trip to Ghana. Our group of 13 had embarked on the long journey from Chicago to Agbouzme in Ghana, a West African country on the coast of the Gulf of Guinea in the eastern tropical Atlantic Ocean, to visit the International Health and Development Network Mission Hospital. Health-care providers from the United States visit this hospital annually. Dr. Edem Agamah, an oncologist originally from Ghana but presently working in Illinois, established this hospital. Dr. Agamah wanted me to see whether I could contribute to the acute management of children there in my capacity as a pediatric intensivist. Nothing was formalized or outlined. The other medical professionals in our team included an anesthesiologist, an adult intensive care nurse, and an infectious disease specialist.

Dr. Blasu is the senior physician in this hospital in Ghana. He is a true general practitioner: rounding in all three areas– pediatrics, adult, and maternity wards; managing the emergency room and clinics; and conducting minor surgeries plus C-sections. There were two other doctors and a couple of physician assistants. First, I rounded on the pediatric floor with them. A nurse carried a bottle of hand sanitizer and came forward to pump a little into our hands every time we touched a patient. Most patients had malaria, and a few had cerebral malaria. They followed the WHO guidelines, a common strategy used to treat most conditions in low-resource nations. There was one child with sepsis. Being a pediatric intensivist, I focused on mental status and urine output. They did not document intake and output there. There was one pulse oximeter, no blood pressure instruments, and not even pediatric nasal cannulas. Nevertheless, the outcome was good.

I then went to the clinic. Three of us, who were visitors, were in one clinic room with a very efficient nurse, Sandra, who interpreted for us. I saw acute conditions: upper respiratory infections, mild bronchiolitis, some diarrhea with mild dehydration, and some fever of unknown origin that inevitably turned out to be malaria.

But again, not everything was easy. All we could offer a young single mother for her malnourished hungry twins was nutrition advice. She was hoping for a medicine to help them gain weight. They clearly were suffering from lack of food. She said she would take a little cereal, mix it with water in a bottle, and let them have half bottle each. That is all she could afford.

The hospital did some charity service, but many people who could not pay got turned away. The realization that this story repeats itself all the time in developing countries is an integral part of global health learning and experiences. In this hospital in Ghana, sometimes there was not enough money to pay the staff, who were already working for less than what they would normally get. The electricity was almost disconnected one time because of a delay in payment. The hospital was doing its best to treat as many patients as it could. But it was difficult, with no sustainable means of income.

The hospital staff tried to do what they could for people, but even if it was not for lack of money, legally there was also nothing they could do to help a resigned, suffering little 8-year-old girl. Grandma said she saw blood spots in her underwear. The girl, very small for her age, had been living with her father. I saw what I was praying I would not see. There were fresh blood at the vaginal orifice and yellow discharge covering most of her genitalia. Apparently, the hospital staff could not call the police in Ghana for such cases of abuse. Grandma would have to make the report. Once she started to change her story, we knew this would not happen. She refused admission, saying she had no money. We offered her oral antibiotics and could only hope that the girl would not be touched again and would receive enough love from her grandmother that she would slowly heal.

Even though I have experience working in low-resource settings, this medical trip to Ghana was still very difficult. At our nightly debriefs, I could sense the same frustration among my teammates “What am I doing here, am I making a difference?” We were experiencing emotions that are not uncommon on medical trips–a sense of helplessness, confusion with the cultural differences, frustration with unfamiliar medical practices, and disheartenment from the lack of resources.

Other major ethical concerns during short-term medical trips include practicing beyond one’s scope of expertise and using scarce resources that can be a drain to local communities. I am a pediatric intensivist, and here I was working in an outpatient clinic in Ghana. Even though I am a pediatrician, I have not worked in an outpatient clinic in over a decade. The local providers could take care of these clinical patients better than I could, and more efficiently. In addition, Sandra, the nurse, had to interpret for me when she could have been better used working where she normally worked, to improve patient flow and management. I quickly realized that I was being a burden to them, using resources that they could make better use of.

The other members of the visiting team were facing similar issues. The critical care nurse and anesthesiologist were frustrated because of unfamiliar practices in Ghana. The relaxed almost laissez faire culture exhibited by the local staff was difficult for these critical care staff to comprehend. They wanted to make several changes to the daily activities there. Although well-intentioned, this attitude could lead to more harm, leaving both host and visitors disenchanted and in a worse position than before.

Our team coped through open discussions. We knew we caused a burden and we wanted to provide something new that would make our trip worthwhile, for us and for our Ghanaian hosts. Therefore, we decided to make a conscious effort to try to empower the local community, value their perspective, and listen to their needs, instead of trying to do what they already did so well. We decided to figure out how we could augment and support them with the expertise we brought with us. We realized that they were saving lives for years with very little resources and that they did not need our criticisms.

Training local providers is one way experts on short medical trips can help improve health-care delivery. The staff in Ghana were educated problem-solvers. From my rounds in the pediatric ward, I realized pediatric critical care assessment skills may be beneficial to help the staff recognize critical illness earlier. We held a seminar which was well attended by doctors and nurses from the region. Each member of the visiting team discussed a few topics each. I talked about how to assess a critically ill child: the discussion included signs of decreased perfusion. I emphasized that mental status and urine output are important indicators of illness in addition to heart rate, temperature, and work of breathing. We also discussed early management of sepsis and that early fluid resuscitation and antibiotics could prevent rapid deterioration and death. The infectious disease specialist in our group discussed diagnosis and management of hepatitis B and C, and HIV. Hepatitis causes greater mortality in Ghana than HIV. There was a lot of interest and discussion on these topics. The anesthesiologist assessed the situation in the operating room and decided to focus on training local staff on preoperative assessment and the concept of “time out” for ensuring accurate patient identity, surgical site, and planned procedure. She created a STOP sign and placed it outside the door of the operating room so that staff would be reminded to perform this before entering.

Longitudinal relationship with host communities, including help with resource development and training, is probably the most effective strategy for improving global health as this would improve care to thousands of people. However, short-term trips, if performed with the best intentions, can be life changing to some individuals who would otherwise never get the service that could dramatically improve their quality of life, including simple orthopedic procedures, ophthalmologic minimally invasive surgeries, and even dental procedures. The hospital in Ghana did not have an anesthesiologist. Because we had one in our team, Dr. Agamah invited a general surgeon from Accra to perform surgeries needing general anesthesia. Forty surgeries were scheduled. Several local people benefited from this, and, to some, this was probably the only opportunity to get the help they needed.

Then, another opportunity opened up for me. A 4-month-old baby was in severe respiratory distress and very lethargic. They wanted to see whether there was something I could do to help them before they endotracheally intubated and bag ventilated him all the way to Accra, almost 3 hours away. A couple of students in our team, Dr. Blasu, and I had been working on improvising a way to deliver bubble continuous positive airway pressure (CPAP). We had just built a prototype with some cannulas (albeit adult), an empty saline bottle, some tape, and a ruler. I started the baby on bubble CPAP, placed him on continuous albuterol (which they had never performed before). Dr. Blasu promised to keep a close eye on him throughout the night. The next morning, I visited the hospital. I saw the beautiful and smiling mother with a cooing baby in her arms. It was almost a miracle. Finally, I felt that, maybe, I had shown them something they could sustain: a change in the way they could manage their little patients. I keep in touch with Dr. Blasu. He recently informed me that they have used this improvised bubble CPAP on a few neonates after we left Ghana and found it very helpful.

I believe that short-term medical trips to resource-limited regions are motivated foremost by a desire not only to help but also to provide a positive experience for everyone involved. However, there are very real risks to both volunteers and host communities. Volunteers may experience frustration, feelings of inadequacy and helplessness, and may risk their personal health. Host communities may suffer from the volunteers competing with local services, using limited resources, donating unnecessary equipment, and overall harming the patients. We were not immune to these difficulties, but we attempted to overcome them by reflection, group support, evaluation of activities and sustainability issues, our own abilities and experience, and partnership with local staff in Agbouzme. We evaluated the needs of the local community and the unique skill sets we brought with us. We attempted to augment the local providers’ skills while respecting and learning from their skills and knowledge of their own community.

